# Outcomes of Delayed Sternal Closure in Pediatric Heart Surgery: Single-Center Experience

**DOI:** 10.1155/2018/3742362

**Published:** 2018-04-19

**Authors:** Daniel Hurtado-Sierra, Juan Calderón-Colmenero, Pedro Curi-Curi, Jorge Cervantes-Salazar, Juan Pablo Sandoval, José Antonio García-Montes, Antonio Benita-Bordes, Samuel Ramírez-Marroquin

**Affiliations:** ^1^Department of Paediatric Cardiology, Instituto Nacional de Cardiología Ignacio Chávez, Mexico City, Mexico; ^2^Department of Pediatric Cardiac and Congenital Heart Disease Surgery, Instituto Nacional de Cardiología Ignacio Chávez, Mexico City, Mexico

## Abstract

**Background:**

Delayed sternal closure (DSC) after cardiac surgery is a therapeutic option in the treatment of the severely impaired heart in pediatric cardiac surgery.

**Methods:**

A single-center retrospective review of all bypass surgeries performed over a 10-year period (2003–2012).

**Results:**

Of a total of 2325 patients registered in our database, the DSC group included 259 cases (11%), and the remaining 2066 cases (89%) constituted the control group (PSC). RACHS-1 risk was higher for the DSC group (74% had a score of 3 or 4) than for the PSC group (82% had a score of 2 or 3). The most frequent diagnosis for the DSC group was transposition of the great arteries (28%). We found out that hemodynamic instability was the main indication observed in patients aged ≤ 8 years (63%), while bleeding was the principal indication for patients aged ≥ 8 years (94%) (*p* ≤ 0.001). The average time between surgery and sternal closure was 2.3 ± 1.4 days. Overall mortality rates were higher for patients of the DSC group (22%) than for the PSC group (8.7%) (OR: 0.4 (95% CI: 0.4 to 0.5), *p* < 0.05). There were six patients with DSC who developed mediastinitis (2.3%). The risk of mediastinitis was significantly higher when DSC was performed 4 days after the primary surgery.

**Conclusions:**

DSC is an important management strategy for congenital cardiac surgery in infants and children. The prolonged sternal closure time is associated with an increased rate of postoperative mediastinitis.

## 1. Introduction

Developed in the 1980s, delayed sternal closure (DSC) in the pediatric population continues to be a useful tool for managing certain groups of patients with congenital heart disease. It is most frequently indicated in complex cases, which require prolonged time of cardiopulmonary bypass (CPB) and aortic cross clamping that induce myocardial edema and predispose to intraoperative bleeding. In this context, DSC, inotropes, and mechanical ventilation are important factors to consider in life support management, in order to achieve appropriate patient survival [1–8].

There is no consensus in the literature regarding the criteria used for indicating DSC, and different clinical series report variable morbidity and mortality rates related to this procedure [9–11]. The aim of this paper is to present our 10-year institutional experience in patients up to 18 years of age who were operated on for congenital heart disease, in order to compare those who received DSC with those who did not, in terms of morbidity and mortality related to the procedure.

## 2. Patients and Methods

### 2.1. Study Design

A retrospective study was designed, in order to include all patients up to 18 years of age who were admitted to the Department of Paediatric Cardiology of our institution for congenital heart disease surgical treatment in a 10-year period (from January 2003 to December 2012). The patients enrolled from our electronic database were divided into two groups: the first one included the cases in whom DSC was performed in the early postoperative period (DSC group), and the second one included those who received a primary sternal closure (PSC group). Exclusion criteria were congenital heart disease operated on without the use of CPB, absence of clinical follow-up in the early postoperative period, and reoperations in the early postoperative period that were not handled with DSC in the primary operation. The variables considered for comparative analysis in both groups were cardiovascular diagnosis, preoperative clinical functional class (according to NYHA/Ross classification), surgical risk (according to RACHS-1 score), CPB and aortic cross clamping time, delayed sternal closure indication, time elapsed until the final sternal closure, and morbidity and mortality associated with the DSC procedure. The early postoperative period was defined as the time between primary surgery for congenital heart disease and 30 days later, or until hospital discharge. Comparison between the two studied groups was done in the pre-, trans-, and early postoperative periods, with emphasis on morbidity and mortality related to DSC.

### 2.2. DSC Surgical Technique

All patients enrolled in this study underwent a cardiovascular anesthesia induction protocol with fentanyl and maintenance with inhaled isoflurane (0.5%), associated with intermittent doses of fentanyl. Congenital heart disease surgery with CPB was performed with a basal rate of 2.5 l/min-m^2^. All patients received 1-2 mg/kg hydrocortisone doses at the anesthesia induction period, as well as 100 mg/kg of cephalothin for antibiotic prophylaxis. Total longitudinal median sternotomy was the classic cardiac surgical approach for all patients. Myocardial protection was performed with hypothermia and crystalloid cardioplegia (Custodiol®), and modified ultrafiltration was performed in all cases. In all patients of the PSC group, after decannulation and appropriate hemostasis, we proceeded to perform a partial pericardial closure and a primary sternum closure with separated surgical metallic sutures, followed by a conventional soft tissue and skin closure.

Indications for DSC were either hemodynamic instability when attempting sternal closure (persistent hypotension, heart rhythm disturbances, increased pressure in the left atrium, or severe decrease in cardiac output) or incoercible bleeding (inability to achieve adequate hemostasis). The surgical technique for patients who were managed with DSC included the externalization of intracardiac catheters, pacemaker electrodes, and pleural and/or mediastinal drainages distal from the skin incision, with lack of any of these elements through it. The pericardium and sternum remained opened as well as the surgical skin incision approach. After removing the surgical sternal retractor, a polyvinylchloride membrane with the shape and size of the skin incision was tailored. This membrane was sutured to the skin edges with a continuous monofilament nonabsorbable suture. Before covering the wound with gauze and sterile transparent adhesive membrane, a 10% iodine-povidone gel was placed all around the synthetic membrane, just at its junction level with the skin.

All the patients of this group were taken to the operating room for definitive sternal closure once the cause of DSC had been controlled. Criteria used to perform a definitive sternal closure included hemodynamic stability in the last 24 hours, neutral to negative fluid balance, normal coagulation status, and lack of acidosis. The surgical technique for definitive sternal closure included removal of the skin sutures and the synthetic membrane itself, as well as removal of fibrin and/or mediastinal clots, which were routinely sent to culture (including a fragment of the synthetic membrane). After washing of the wound with diluted iodine-povidone solution, including sternal tables and pericardial cavity, partial closure of the pericardium was performed, followed by sternal closure with separated surgical metallic sutures. Finally, a conventional soft tissue and skin closure was made, as described for the PSC group.

### 2.3. Statistical Analysis

Data obtained from medical records were compiled in an Excel® spreadsheet and then processed using the SPSS statistical software v.21.0 (SPSS Inc., Chicago, IL). Categorical variables are presented as frequency and percentage in relation to the population at risk. Continuous variables are presented as mean ± standard deviation and minimum and maximum ranges of variability. For variable comparison between the two groups of study, Pearson's Chi-square test was used with an estimated 95% confidence interval odds ratio. For comparison of continuous data, Student's* t*-test or a Mann–Whitney* U* test was used, as required. The intersection of age values due to the cause of DSC was performed by plotting logarithmic trend curves. *P* values < 0.05 were considered statistically significant.

## 3. Results

We enrolled 2325 cases out of a total of 4057 patients registered in our electronic database in the study period. The DSC group included 259 cases (11%), and the remaining 2066 cases (89%) constituted the control group (PSC). The rest of the patients were not considered for this study because cardiac surgery was performed by means of a thoracotomy approach. Differences between both studied groups are discussed at the pre-, trans-, and early postoperative periods as follows.

### 3.1. Demographic and Clinical Variables Comparison at the Preoperative Period

Table 1 shows demographic and clinical characteristics of both studied groups at the preoperative period. There were more female patients in the PSC group than in the DSC group (50.8% versus 38.6%, *p* < 0.005). Mean age was lower in those who received a DSC procedure (2.8 ± 4.7 years) than in the PSC group (5.3 ± 5.1 years). The requirement of inotropic drugs at the preoperative period was higher in the DSC group than in the PSC group (22.4% versus 4.6%, *p* < 0.05), as was the use of mechanical ventilation before the surgical procedure (25.5% versus 5%, *p* < 0.05). Operative RACHS-1 risk was higher for the DSC group (74% had a 3 or 4 score) than for the PSC group (82% had a 2 or 3 score).  Table 2 shows that the most frequent diagnoses for the DSC group were transposition of the great arteries (28.8%), total anomalous pulmonary vein connection (17%), double outlet right ventricle (10.5%), tetralogy of Fallot/pulmonary atresia with ventricular septal defect (8.9%), valve disorders (5.8%), and univentricular heart (4.6%).

### 3.2. Comparison of Variables at the Trans- and Early Postoperative Period

As shown in Table 1, cardiopulmonary bypass and aortic cross clamping times were longer for the primary surgical procedure in the DSC group than in the PSC group (163.7 versus 98.5 min, *p* < 0.05, and 96.7 versus 56.9 min, *p* < 0.05, resp.).

**Figure 1:**
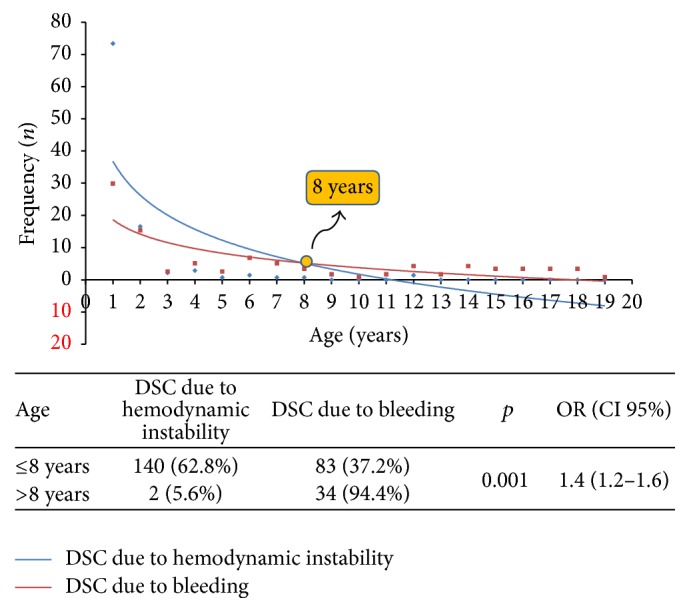
Frequency trends plotted according to DSC indication.

Figure 1 shows the logarithmic age frequency trends plotted for the type of DSC indication. We can see a clear intersection at the age of 8 years. Taking this age value as a reference for analyzing the DSC indication type, we found out that hemodynamic instability was the main indication observed in patients aged ≤8 years (62.8%) while bleeding was the main indication observed for patients aged ≥ 8 years (94.4%), with a statistically significant difference [*p* ≤ 0.001, OR 1.4 (1.2–1.6)]. The average time between primary surgery and definitive sternal closure was 2.3 ± 1.4 days (range: 1–9 days). Most of the patients underwent definitive sternal closure 2 days after primary surgery.

Overall mortality rates were higher for patients in the DSC group (22.4%) than for those in the PSC group (8.7%), with a statistically significant difference (OR: 0.4 (95% CI 0.4 to 0.5), *p* < 0.05). Patients with a RACHS-1 score ≥ 3 had a higher mortality risk with DSC than with PSC. A nonclassifiable RACHS-1 score (NC) did not show a significant difference in mortality rate either with or without DSC (Tables 1 and 3).

There were six patients with DSC who developed mediastinitis in this series (2.3%), but none of them died. The risk of mediastinitis was significantly higher when DSC was performed 4 days after the primary surgical operation [OR: 0.039 (95% CI: 0005–0328, *p* < 0.05)], as shown in Table 4.

## 4. Discussion

Heart compression by adjacent structures can reduce end diastolic volume of both ventricle chambers, which may lead to hemodynamic instability due to decreased cardiac output. This pathophysiological situation is worse when talking about cardiopulmonary bypass surgery in young children, because myocardial and pulmonary edema may lead to even greater thoracic restriction. In 1975, Riahi et al. [1] were the first to emphasize the importance of heart/mediastinum mismatch at the postoperative period of cardiac surgery and to propose DSC as a helpful technique for minimizing the effects of the pathophysiological cardiac tamponade-like scenario that it triggers [14, 15]. Actually, palliative and corrective surgery for complex congenital heart disease requires longer times of cardiopulmonary bypass and aortic cross clamping, which may produce negative effects on myocardial function. A significant increase in heart size causes ventricular dysfunction and important myocardial perfusion disorders when trying to close the chest. Therefore, DSC has become a helpful technique for achieving earlier recovery and better results in pediatric patients with congenital heart disease [2, 14, 15]. Indications for use of DSC depend on several factors, but age is one of the most important ones. Tabbutt et al. [11], in a 4-year retrospective study that included patients up to 25 years old, reported a frequency of 8.4% for DSC. Alexi-Meskishvili et al. [12], in a retrospective analysis of patients up to 18 years old, reported a frequency of 9% for DSC. These studies contrast with reports in newborns, where the DSC rate is higher. Samir et al. [6], in a 10-year retrospective study, reported a DSC rate of 45%, and McElhinney et al. [7] reported a DSC rate of 22% in a 7-year study. The higher frequency of DSC at the neonatal period can be attributed to a substantial increase of thoracic pressure because of high peak pressures required for poor distensible lung ventilation due to pulmonary edema. In this series, we found an 11% overall frequency of DSC (Table 5) and a DSC frequency of 55% for the ≤6 months' age group. Similarly, 70% of the patients were managed with DSC in the study of Alexi-Meskishvili et al. [12] which included neonates and infants up to 6 months old. These findings were also observed in other reported series [11, 14].

Transposition of the great arteries and hypoplastic left heart syndrome were the main diagnoses of the patients who were managed with DSC in most series [4–6, 11, 12, 14]. In our study, transposition of great arteries represented 28% of all diagnoses, followed by total anomalous pulmonary venous connection (17%). We must state that the number of patients annually diagnosed with hypoplastic left heart syndrome at our institution is relatively low, representing only 1.9% of all patients with DSC (Table 2). There are several indications for DSC in cardiovascular surgery, but they can mainly be summarized in two groups according to their pathophysiological mechanism: hemodynamic instability and uncontrollable bleeding. In studies that included only neonates, the main cause that led to DSC was hemodynamic instability. Tabbutt et al. [11], who studied patients of all ages, stated that the decision to use DSC in the immediate postoperative period was taken electively before the procedure in 25% of cases and due to hemodynamic instability in 37% and bleeding in 7%. These results contrast with those reported by Riphagen et al. [5], where the main cause of DSC was bleeding in 50% of cases, followed by hemodynamic instability in 24%. In our series, we observed that hemodynamic instability was the main cause for DSC in the group of neonates and infants. In contrast, DSC due to bleeding was found in patients above 8 years of age. We attribute this age-significant difference in the indication of DSC to a greater probability of bleeding due to reoperation in patients above 8 years of age when facing the surgical treatment of their complex heart disease [16]. Additionally, it is important to highlight that there is also a delayed reference of several patients with complex heart disease for surgical treatment at our institute. This fact explains the reason for performing a high number of DSC in this population, which is quite different from that of many other teams reported in the literature.

There is a consensus that indications for delayed sternal closure are mainly to mitigate poor cardiac output and allow for resolution of inflammation and edema of the myocardium and lungs and/or to facilitate mediastinal exploration in patients with incomplete hemostasis. Despite the several literature publications, controversies remain regarding criteria used to perform the definitive sternal closure. The optimal time for definitive sternal closure is not yet clearly defined because there are several criteria stated by different institutions and healthcare centers, such as the need for achieving a negative water balance as well as hemodynamic stability [2, 11, 12, 14–18]. Riphagen et al. [5] suggest that definitive sternal closure may be performed at an average time of 21 hours (range: 18–40 hours). They mention that this is the time needed to observe the effects of cardioprotective steroids used before cardiopulmonary bypass, modified ultrafiltration, early use of phosphodiesterase inhibitors, and short cardiopulmonary bypass (average of 84 min). In this series, great part of our patients remained with an opened sternum for 2 days, which was the time usually required to control hemodynamic instability or bleeding. We must highlight the fact that although we had a longer time of cardiopulmonary bypass compared with that reported by Riphagen et al. [5], our time between primary surgery and definitive sternal closure was significantly lower than that reported by other series (Table 5). DSC has been considered as a risk factor for developing mediastinitis. However, there is still no consensus about the real risk that patients with DSC have for the development of such a complication [19–21]. Literature review shows that the incidence of mediastinitis due to DSC ranges from 1.8% to 5.6% [22]. There were six cases of mediastinitis out of 259 patients with DSC (2.3%) in our series. In this study, we found that there is a statistically significant risk for developing mediastinitis when definitive sternal closure following DSC is performed after 4 days [OR: 0.039 (95% CI 0.005 to 0.328, *p* < 0.05)]. Nelson-McMillan et al. [23] reported a 14% to 16% risk of infection complication rate up to the sixth day after delayed sternal closure, which increases dramatically to 35% from the seventh day onward (OR = 3.87). Finally, we consider, like Al-Sehly et al. [22], that the risk of developing mediastinitis due to DSC is low and does not contraindicate the use of this technique (Table 4). Tabbutt et al. [11] reported a 19% mortality rate due to DSC in a significant number of patients, as did Özker et al. [4], who reported a rate of 34.2%. The mortality rate due to DSC of our series was 22.4%. Additionally, we found that the mortality risk is higher in patients with DSC than in the PSC group [OR = 0.4 (95% CI 0.4 to 0.5), *p* < 0.05]. Interestingly, all patients with a RACHS-1 score ≥ 3 have a higher risk of mortality with DSC than those patients with PSC (Table 3).

## 5. Conclusions

Based on the data analysis of this study, we found that DSC was most used in patients who had preoperative inotropic and mechanical ventilatory support. Other risk factors for DSC were prolonged time of cardiopulmonary bypass and aortic cross clamping. Surgical indications for DSC are related to the patient's age, because the main indication in younger patients is hemodynamic instability, whereas the main indication in older patients (≥8 years old) is bleeding. There was a statistically significant risk of mediastinitis when definitive sternal closure was performed after 4 days. This study suggests that the DSC is a tool that should be used judiciously and suggests proceeding to definitive sternal closure when the hemodynamic stability of the patient allows it.

## Figures and Tables

**Table 1:** Demographic and clinical characteristics of the study groups.

Variable	PSC (*n* = 2066)	DSC (*n* = 259)	*p*	OR (CI 95%)
Age (years)	5.3 ± 5.1	2.8 ± 4.7	0.001	
Female gender	1049 (50.8%)	100 (38.6%)	0.001	0.6 (0.5–0.8)
Preop inotropic use	95 (4.6%)	58 (22.4%)	0.001	0.2 (0.19–0.31)
Preop mechanic ventilation	104 (5%)	66 (25.5%)	0.001	0.2 (0.18–0.3)
Surgical RACHS-1 risk score				
1	264 (12.8%)	1 (0.4%)	0.001	33.2 (4.7–235.5)
2	1082 (52.4%)	48 (18.5%)	0.001	4.1 (3.07–5.62)
3	590 (28.6%)	103 (39.8%)	0.001	0.6 (0.5–0.8)
4	120 (5.8%)	89 (34.4%)	0.001	0.2 (0.1–0.2)
5	0 (0%)	3 (1.2%)	0.001	0.1 (0.09–0.1)
6	3 (0.1%)	14 (5.4%)	0.001	0.1 (0.09–0.1)
NC	7 (0.3%)	1 (0.4%)	0.001	
CPB time (min)	98.5 ± 53.8	163.7 ± 62	0.001	
ACC time (min)	56.9 ± 41	96.7 ± 54.5	0.001	
Mortality	180 (8.7%)	58 (22.4%)	0.001	0.4 (0.3–0.5)

CPB: cardiopulmonary bypass; ACC: aortic cross clamp; PSC: primary sternal closure; DSC: delayed sternal closure; NC: nonclassifiable; Preop: preoperative.

**Table 2:** Congenital heart disease diagnosis of the DSC group.

Diagnosis	*n*	%
TGA	72	28
TAPVC	44	17,1
DORV	27	10,5
T. Fallot/PA with VSD	23	8,9
Valve disorders	15	5,8
Truncus arteriosus	14	5,4
Univentricular heart	12	4,6
VSD	6	2,3
Aortic coarctation	9	3,5
Pulmonary valve agenesis	6	2,3
Complete A-V canal	5	1,9
Hypoplastic left heart syndrome	5	1,9
Congenitally corrected TGA	4	1,6
PA without VSD	4	1,6
Other	13	4,6

*Total*	*259*	*100*

TGA: transposition of great arteries; TAPVC: total anomalous pulmonary venous connection; DORV: double outlet right ventricle; T. Fallot: tetralogy of Fallot; PA: pulmonary atresia; VSD: ventricular septal defect.

**Table 3:** Comparison between the RACHS-1 preoperative surgical risk score and the overall mortality.

RACHS -1	PSC	DSC	*p*	OR (CI 95%)
3	590 (28.6%)	103 (39.8%)	0.001	0.6 (0.5–0.8)
4	120 (5.8%)	89 (34.4%)	0.001	0.2 (0.1–0.2)
5	0 (0%)	3 (1.2%)	0.001	0.1 (0.09–0.1)
6	3 (0.1%)	14 (5.4%)	0.001	0.1 (0.1–0.16)
NC	7 (0.3%)	1 (0.4%)	0.612	

PSC: primary sternal closure; DSC: delayed sternal closure.; NC: nonclassifiable; RACHS-1: Risk Adjustment in Congenital Heart Surgery.

**Table 4:** Comparison between the time at definitive sternal closure and the risk of mediastinitis due to DSC.

Time at DfSC (days)	Mediastinitis due to DSC		*p*	OR (CI 95%)
	Yes	No		
<4 days	1 (16.7%)	197 (85.3%)	0.001	0.039 (0.005–0.328)
≥4 days	5 (83.3%)	34 (14.7%)		

DfSC: definitive sternal closure; DSC: delayed sternal closure.

**Table 5:** Comparison between published series related to DSC at the last decade literature review.

Series	Age of the studied group	Period	DSC/CPB	Percentage (%)	Mortality (%)	Time at DfSC (days)
Current study	<18 years	2003–2012	257/2347	11%	22.4%	2.3^*^
Alexi-Meskishvili et al. [12]	<18 years	1990–2003	113/1252	9%	36%	5^*^
Iyer et al. [13]	Neonates and infants	1986–1995	150/3718	4%	11%	3.9^*^
Elami et al. [14]	Neonates and infants	1987–1992	36/641	5.6%	5.6%	5^*^
Özker et al. [4]	Neonates and infants	2007–2011	38/1011	3.45%	34.2%	2.9^*^
Riphagen et al. [5]	Neonates and infants	2000–2003	66/585	11.2%	20%	0.9^*^
Samir et al. [6]	Neonates	1991–2000	140/312	44.8%	21%	-
McElhinney et al. [7]	Neonates and infants	1991–1996	128/585	21.8%	21%	3^**^
Tabbutt et al. [11]	<18 years	1992–1995	217/2559	8.4%	26%	3.4^**^

CPB: cardiopulmonary bypass; DSC: delayed sternal closure; DfSC: definitive sternal closure; ^*^mean value; ^**^median value.
